# Mortality Prediction of Septic Patients in the Emergency Department Based on Machine Learning

**DOI:** 10.3390/jcm8111906

**Published:** 2019-11-07

**Authors:** Jau-Woei Perng, I-Hsi Kao, Chia-Te Kung, Shih-Chiang Hung, Yi-Horng Lai, Chih-Min Su

**Affiliations:** 1 Department of Mechanical and Electro-Mechanical Engineering, National Sun Yat-sen University, Kaohsiung 804, Taiwan; jwperng@faculty.nsysu.edu.tw (J.-W.P.); flush0129@gmail.com (I.-H.K.); 2 Department of Emergency Medicine, Kaohsiung Chang Gung Memorial Hospital, Chang Gung University College of Medicine, Kaohsiung 83301, Taiwan; g00308@cgmh.org.tw (C.-T.K.); hsc0901@cgmh.org.tw (S.-C.H.); 3 School of Mechanical and Electrical Engineering, Xiamen University, Tan Kah Kee College, Zhangzhou 363105, China; lai81.tom@gmail.com

**Keywords:** deep learning, machine learning, mortality prediction, neural networks, sepsis

## Abstract

In emergency departments, the most common cause of death associated with suspected infected patients is sepsis. In this study, deep learning algorithms were used to predict the mortality of suspected infected patients in a hospital emergency department. During January 2007 and December 2013, 42,220 patients considered in this study were admitted to the emergency department due to suspected infection. In the present study, a deep learning structure for mortality prediction of septic patients was developed and compared with several machine learning methods as well as two sepsis screening tools: the systemic inflammatory response syndrome (SIRS) and quick sepsis-related organ failure assessment (qSOFA). The mortality predictions were explored for septic patients who died within 72 h and 28 days. Results demonstrated that the accuracy rate of deep learning methods, especially Convolutional Neural Network plus SoftMax (87.01% in 72 h and 81.59% in 28 d), exceeds that of the other machine learning methods, SIRS, and qSOFA. We expect that deep learning can effectively assist medical staff in early identification of critical patients.

## 1. Introduction

Sepsis is a disease with various presentations and a high mortality rate, making it difficult for doctors to evaluate all clinical data to accurately assess these patients. This problem is particularly challenging in the emergency department (ED).

Systemic inflammatory response syndrome (SIRS) and quick sepsis-related organ failure assessment (qSOFA) are simple methods that allow for the assessment of sepsis. Both have been used for years; however, the debate as to whether qSOFA or SIRS is the superior method has continued, as both approaches lack sensitivity and specificity [1,2,3,4].

In recent years, various artificial intelligence (AI) applications have been gradually implemented in the medical field by using machine learning [5,6,7], resulting in more accurate results. There are two types of machine learning: unsupervised learning and supervised learning. There are many applications for unsupervised learning, including principal component analysis (PCA) [8,9], K-means algorithms [10], and self-organizing maps [11]. Similarly, there are also many supervised learning algorithms that have been applied to solve several engineering problems, including support vector machines (SVMs) [12,13], artificial neural networks [14], and partial least squares [15]. Few studies have compared their accuracy in medical prediction models, and there have been no studies to date in which combining feature extraction and classification of machine learning models has been used to increase discrimination ability.

In our study, various machine learning algorithms were introduced to create a better predictive model for identifying patients who are most at risk for sepsis. The accuracy rates for predicting mortality at 72 h and 28 d, as well as area under curve of receiver operating characteristic (AUC), were compared with each other and qSOFA and SIRS.

## 2. Methods

### 2.1. Study Design

We report herein a retrospective study of ED patients who were suspected of having infection. We collected variables from these patients and used different machine learning algorithms to predict mortality over 72 h and 28 d in hospital. This study was approved by the institutional review board with waiver of informed consent (IRB number 103-0053B).

### 2.2. Study Setting and Population 

The data were obtained from Chang Gung Research Database between January 2007 and December 2013. Kaohsiung Chang Cheng Memorial Hospital, a 2692-bed acute-care teaching hospital, is the largest medical center in Southern Taiwan providing both primary and tertiary referral care. All adult patients admitted from the ED that had blood culture collected and received intravenous antibiotics were enrolled in the study. Patients under 18 years of age, and those who were discharged within three days of admission, were excluded from the study, in addition to those that did not have more than 10 clinical data points required by our working model.

### 2.3. Dataset Creation and Definition

Only those data collected during the ED visit, and up until the time of admission, were used as prediction variables. There were 53 clinical variables that were chosen as factors for the machine learning algorithms. These variables, listed in Table A1, were all clinically meaningful, and were divided into three categories: demographic data, vital signs, and laboratory results. Table A1 also shows the distribution of these variables among 28-day survivors and non-survivors. ICD-9 codes [16] for past medical history, ED clinical impression, and hospital discharge diagnoses were recorded. Shock episode was defined as administration of inotropic agents, including dopamine and norepinephrine, during ED admission. The dose and timing of all parental antibiotics administered in the ED was also recorded.

Total and white blood cell counts were measured by Sysmex XE 500. The serum biochemistry parameters, including BUN, creatinine, sodium, potassium, aspartate aminotransferase, alanine aminotransferase, and Troponin I, were assessed using a Uni Cel DX 880i, while prothrombin time and activated partial thromboplastin time were measured by the Sysmex CS 2100i.

SIRS is determined using four indicators. The first indicator is that “body temperature must be lower than 36 °C or higher than 38 °C”. The second indicator is that the “heart rate of patient must be greater 90 beats/min”. The third indicator is that the “respiratory rate of patients must be lower than 20 breaths/min”. The last indicator is that the “abnormal white blood cell count must lower than 12 × 109 or greater than 4 × 109/µL or higher than 10% immature (bands) forms”. One point was assigned when each of the above conditions was observed, with scores ranging from zero to four [17]. Criteria for SIRS are considered to be met if at least two of the above four clinical findings are present.

The qSOFA score is a total of three indicators. The first indicator is that “systolic blood pressure must be lower than 100 mmHg”. The second indicator is that “the respiratory rate must be greater than 22 breaths/min”. The last indicator is that “Glasgow Coma Scale (GCS) must be greater than 13”. One point was assigned when each of the above conditions was observed, with the resulting scores ranging from zero to three [18]. Thus, for both SIRS and qSOFA, a higher score indicates a more serious condition. Criteria for qSOFA are considered to be met if at least two of the above three clinical findings are present.

### 2.4. Data Processing

Patients who were under 18 years of age were excluded. Patients under the age of 18 should be included in the scope of pediatric exploration. Patients discharged from the emergency department within 72 h or 28 d were excluded because their outcomes were unknown.

The medium number of the column was substituted for missing data. The effect of missing values on the model can be greatly reduced by mean or median replacement and establishing an L1 or L2 constraint in the neural network [19,20]. After the clinical features were chosen, the values of the datasets were normalized between −1 and 1 by standardization. The following equation illustrates standardization of the verification data:

(1) $$z_{i}=\frac{x_{i}-\mu }{\sigma },$$

where $x=\left(x_{1},\;\ldots ,x_{n}\right)$, $z_{i}$ is now the *i*th standardized dataset, μ is the mean of x, and σ is the standard deviation of x.

The testing dataset was 30% of the total dataset and the training dataset was 70% of the total dataset. The datasets of surviving patients and non-surviving patients were also split-averaged. To confirm that the method implemented in this paper resulted in a generalized ability to predict our experimental data, we used K-fold cross validation to conduct the experiments. However, in this paper, the results of only one experiment will be displayed for the sake of brevity.

### 2.5. Outcome

The primary outcome was 72 h and 28 d in-hospital mortality with all causes.

### 2.6. Machine Learning Model

#### 2.6.1. Autoencoder (AE)

An autoencoder (AE) is a type of fully connected neural network that has the ability to perform feature extraction and data compression [21]. AE contains two structures: an encoder and a decoder. The encoder is used to compress the data, and the decoder is used to revert the data. However, this reversion does not result in a dataset that is identical to the input data. The advantage of the AE is that it learns and implements the encoders and decoders on its own and reduces the handcrafted parameters. The encoder and decoder can be described using the following mathematical formula:

(2) $$\begin{array}{c} \phi :x\to F,\\ \psi :F\to x,\\ \phi ,\psi =arg\;{min}_{\phi ,\;\psi }{\left\|x-\left(\psi \circ \phi \right)x\right\|}^{2}, \end{array}$$

where ϕ is the encoder, ψ is the decoder, x is the input, F is the output of the encoder, and ∘ is a function composition. In a simple case, there is a hidden layer. The following formula is used as the input of the encoder.

(3) $$x\in {\mathbb{R}}^{d}=X,$$

and it is mapped to

(4) $$z\in {\mathbb{R}}^{p}=F.$$

The following formula can be obtained.

(5) $$z=f_{act}\left(\omega x+\varrho \right),$$

where *z* indicates the features that are being compressed and ω are the weights. After encoding is completed, *z* is reconstructed by the decoder to equalize $\overset{´}{x}$ and x, reverting the input. The described formula is:

(6) $$\overset{´}{x}=\overset{´}{f_{act}}\left(\overset{´}{\omega }z+\overset{´}{\varrho }\right),$$

where the parameters $\overset{´}{\varrho }$, $\overset{´}{\omega }$, and $\overset{´}{f_{act}}$ are not necessarily the same as the encoder.

Using the encoder, the features of the clinical variables can be reduced. A fully connected layer and a SoftMax layer are connected after the encoder. SoftMax is an activation function of backpropagation neural networks. The SoftMax function is essentially the gradient logarithm normalization of the finite discrete probability distribution.

In this research, the 53 clinical variables can have their dimensionality reduced by AE. Therefore, AE was designed as a single-input-multiple-output system to create an end-to-end neural network. One of the outputs was the output of the AE, and another output was the output of the SoftMax layer. The output of the SoftMax released the mortality rate of the patient, which was the goal of this study.

**Figure 1:**
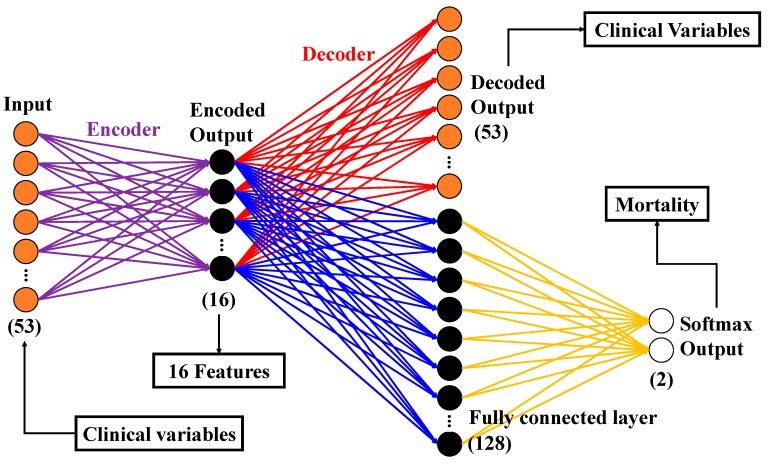
Structure of the autoencoder (AE) in this experiment.

Such a method not only retains a large number of original data features but also achieves the purpose of dimensionality reduction. The structure of AE used in this study is shown in Figure 1.

The activation function of the hidden layer was the rectified linear unit (ReLU) as presented below:

(7) $$f_{ReLU}\left(x\right)=max\left(0,x\right)$$

Further, the activation function of the decoded output was a sigmoid as presented below:

(8) $$f_{sigmoid}\left(x\right)=\frac{e^{x}}{e^{x}+1}$$

Before every activation function, batch normalization was performed [22]. There are several benefits to adding batch normalization to a deep learning structure, such as decreased training time, prevention of gradient vanishing, and minimal overfitting. Nevertheless, there still existed a marginal overfitting in the experimental results after adding batch normalization. Therefore, dropout [23] was also considered while designing the AE structure. To further reduce overfitting, all of the hidden layers of the AE included a dropout rate of 20%.

The input layer and the decoded AE output had 53 neurons, which was the same as the clinical variables. The encoded output had 16 neurons. To perform feature extraction, the hidden layer was the output of the encoder. There were 16 extracted feature dimensions. A fully connected layer with 128 neurons was connected after the encoder output. A SoftMax layer with two neurons was connected after the fully connected layer. The SoftMax layer produced the primary output that we required.

The optimizer for the training experiment was Adam [24] with a learning rate of 0.001. The loss of the training experiment was the mean squared error. The batch size was the number of values in the training dataset. This implies that all training data will be trained within a specified time. The maximum epoch was 10,000; however, we designed a checkpoint and early stop to avoid wasting time. If the loss value did not renew itself with a better value within 100 epochs, the training process would be shut down and the best model with the lowest loss was saved.

#### 2.6.2. Convolutional Neural Network (CNN)

Convolutional Neural Networks (CNNs) are deformed networks derived from artificial neural networks [25]. They are widely used in image processing and video recognition systems [26]. Most of the structures of the CNN are used for 2D training images, but the datasets are in the 1D domain. For these reasons, the structures must be altered into a 1D-CNN [27,28,29].

The advantage of CNN is weight sharing, which reduces the number of parameters that need to be trained. Furthermore, CNN can reduce the number of features, making it easier for these features to be classified.

CNN is based on backpropagation to perform error correction for each layer of weight. The output error of the convolutional layer can be calculated with a pooling layer.

(9) $$\delta^{c}=upsample\left(\delta^{p}\right)⨀\overset{´}{f_{act}}\left(z^{c}\right)$$

where $\delta^{c}$ is the error of the convolutional layer, $\delta^{p}$. is the error of the pooling layer, $z^{c}$ is the output value of the convolutional layer, and ⨀ is the pointwise product. In the pooling layer, no activation functions were set. Using the error of the convolutional layer, the error of the last hidden layer can be calculated:

(10) $$\begin{array}{c} for\;h=c-1,c-2,c-3,\ldots ,2\\ \delta^{h}=\delta^{c}\frac{\partial z^{c}}{\partial z^{h}}=\delta^{c}*rot180\left(\omega^{c}\right)⊙\overset{´}{f_{act}}\left(z^{h}\right) \end{array}$$

where $\omega^{c}$ is the weight of convolutional layer. Here, rot180 implies a 180° rotation of the matrix. From the error of the convolutional layer, the gradient of weight and bias can be calculated:

(11) $$\frac{\partial C\left(\omega ,b\right)}{\partial \omega^{c}}=\frac{\partial C\left(\omega ,b\right)}{\partial z^{c}}\frac{\partial z^{c}}{\partial \omega^{c}}=\delta^{c}*rot180\left(\overset{´}{f_{act}}\left(z^{h}\right)\right)$$

(12) $$\frac{\partial C\left(\omega ,b\right)}{\partial b^{c}}=\sum_{u,v}{\left(\delta^{c}\right)}_{u,v}$$

where *u* and *v* are the sizes of the tensor and *b* is the bias of the neuron.

**Figure 2:**
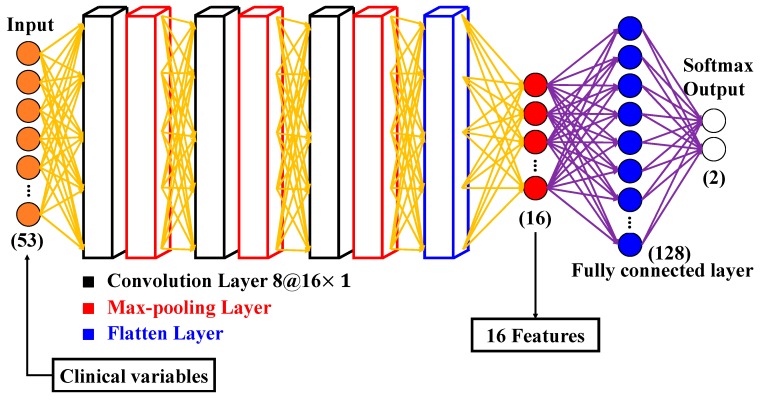
Structure of Convolutional Neural Network (CNN) in this experiment.

In this study, dimensionality reduction for the 53 clinical variables was achieved by CNN, as the number of clinical variables was reduced to 16 vectors. The structure of CNN is shown in Figure 2. There were three convolutional layers in the CNN architecture and each convolutional layer had eight filters at a size of 16×1. The activation function of the convolutional layers is the ReLU. The number of strides for each convolutional layer was set to one. The padding of the convolutional layer was also one. The max-pooling layers were connected after every convolutional layer. The pooling size of the max-pooling layer was two and the number of strides of the max-pooling layer was also two. After all the convolutional layers and max-pooling layers, the neurons were flattened and connected with two fully connected layers. One of the fully connected layers had 16 neurons and the other had 128 neurons. The activation function of the fully connected layers was also ReLU. The fully connected layer with 16 neurons was the output of the feature extraction. A SoftMax layer with two neurons was connected after the fully connected layer. The SoftMax layer presents the primary output that we required, the mortality rate of the patients.

The CNN was combined with SoftMax and trained as an end-to-end structure; however, the network was truncated to extract the data before entering SoftMax. The output was the feature extraction of the CNN. To calculate AUC, the classifier must be a regression method. To achieve this, an operation point was set so that classification could be performed by a regression method.

The CNN training process optimizer was also Adam with a learning rate of 0.001, and the loss of the training experiment was the categorical cross entropy. The batch size was the number of values in the training dataset, and the early stop and checkpoint were the same as the training process for AE.

#### 2.6.3. PCA

PCA is a traditional machine learning algorithm that is often used to reduce features or to perform feature extraction. By using linear transformation, features can be transformed to a new coordinate system. In this new coordinate system, features that have the largest variance are the first dimension, while those with the second largest variance are the second dimension, and so on, with the feature with the smallest variance as the last dimension. Features with the highest variance are usually linearly independent, and it is for this reason that the feature with the greatest variance was chosen for reduction.

#### 2.6.4. Classification Models

There are four classifications of machine learning methods that are often used: K nearest neighbor (KNN) [30], Support Vector Machine (SVM), SoftMax, and Random Forest (RF) [31]. KNN uses the feature space for classification. The input feature is classified using the training features that are around the input features in the feature space. Therefore, while using KNN, the distance of the neighbor is determined by a human. SVM identifies the best hyperplane for classification. The advantage of SVM is that it is stable in managing linear data and is widely used. RF is a structure with more than one decision tree, making it more stable than one decision tree, thereby avoiding underfitting or overfitting. Softmax is an activation function that will never fit the label, resulting in continued training by the neural network until the early stop, or the maximum epoch, is reached.

### 2.7. Statistics

In this study, the coding language used was Python v3.6 and the machine learning platform was TensorFlow v14.1. TensorFlow has a comprehensive, flexible ecosystem of tools, libraries, and community resources, which allow researchers to use state-of-the-art machine learning and developers to easily build and deploy machine-learning-powered applications. The figures in the article were generated by MATLAB (R2018a). Delong test (Medcalc v19.1) was implemented to compare the AUC of the results.

## 3. Results

### 3.1. Patient Management Results

**Figure 3:**
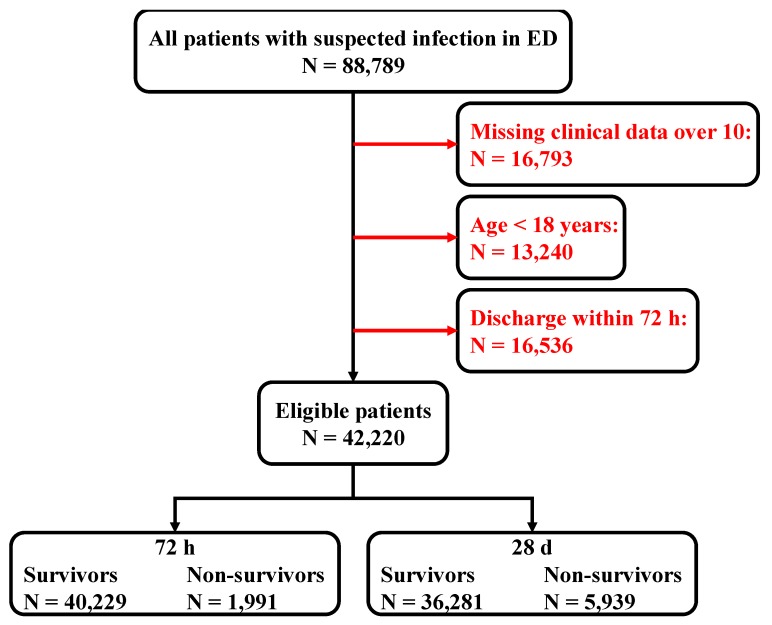
Structure of patient data management.

There were 16,793 patients that were omitted from our study, as they did not meet the requirement of having more than 10 clinical data points, required by our model. Furthermore, there were 13,240 patients under the age of 18 that were also omitted. There were 16,536 patients who were discharged from the emergency room within 72 h that were also not included. Finally, this resulted in 42,220 eligible patients for our study. Of these patients, 1991 died within 72 h, and 5939 died within 28 d. The data cleaning flow chart is shown in Figure 3.

### 3.2. Feature Extraction Results

**Figure 4:**
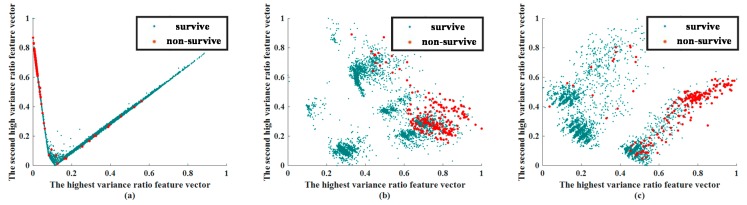
Extracted feature visualization of mortality prediction of testing dataset within 72 h: (**a**) CNN, (**b**) AE, (**c**) PCA. Abbreviation: PCA, principal component analysis; AE, autoencoder; CNN, convolutional neural network.

**Figure 5:**
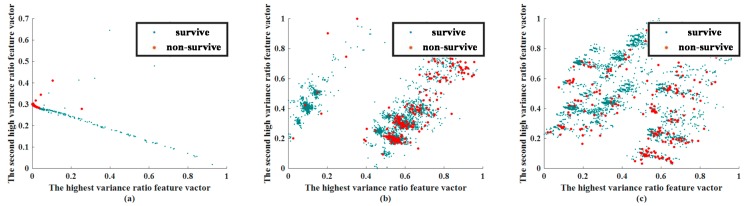
Extracted feature visualization of mortality prediction for the testing dataset in 28 d: (**a**) CNN, (**b**) AE, (**c**) PCA. Abbreviation: PCA, principal component analysis; AE, autoencoder; CNN, convolutional neural network.

In this stage, a visual approach was used to compare the efficacy of CNN and AE in predicting patient outcomes. This experiment was conducted for feature visualization purposes only, so that we could determine whether the applied methods of feature extraction could distinguish between surviving and non-surviving patients. The extracted feature visualization of mortality prediction within 72 h and 28 d are shown in Figure 4 and Figure 5, respectively.

The extracted features of CNN and AE were compared with a traditional feature extraction method, PCA. While reevaluating the results of feature extraction, two phases must be observed. In the visualization of the extracted feature, we chose the features that had the highest variance and second highest variance, and show them in a 2D plane. The values of the feature are scaled by the min–max scale zero to one. In general, if the Euclidean distance between the data can be effectively separated at this stage, the classifier can more easily identify these features.

### 3.3. Classification Results

The experimental classification used four types of classification algorithms—RF, KNN, SVM, and SoftMax—to classify the datasets and the features extracted by the feature extraction algorithms.

**Figure 6:**
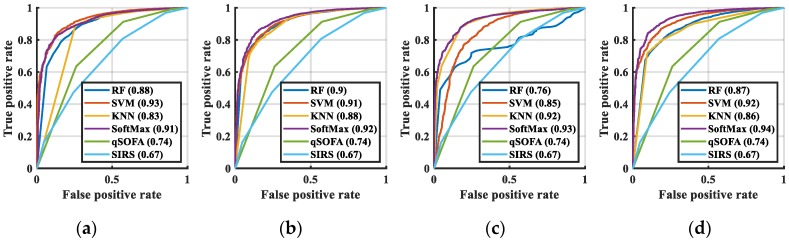
Area under curve (AUC) curve of the mortality prediction for the testing dataset at 72 h: (**a**) no feature extraction, (**b**) PCA, (**c**) AE, (**d**) CNN. Abbreviation: SIRS, systemic inflammatory response syndrome; qSOFA, quick sepsis-related organ failure assessment; RF, random forest; KNN, K nearest neighbor; SVM, support vector machine; PCA, principal component analysis; AE, autoencoder; CNN, convolutional neural network.

**Figure 7:**
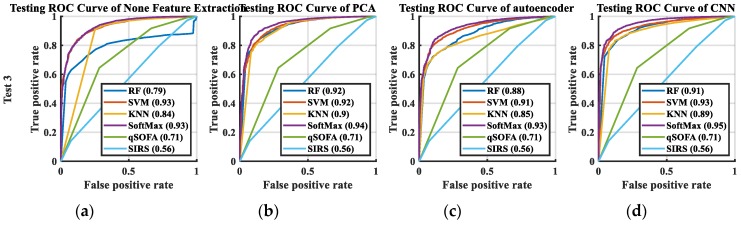
AUC curve of the mortality prediction of the testing dataset in 28 d: (**a**) no feature extraction, (**b**) PCA, (**c**) AE, (**d**) CNN. Abbreviation: SIRS, systemic inflammatory response syndrome; qSOFA, quick sepsis-related organ failure assessment; RF, random forest; KNN, K nearest neighbor; SVM, support vector machine; PCA, principal component analysis; AE, autoencoder; CNN, convolutional neural network.

In this study, two indicators were used to measure the quality of the classification results of the machine learning: the receiver operating characteristic (AUC) curve and the accuracy rate. The AUC curve of the mortality prediction at 72 h is shown in Figure 6, and that at 28 d is shown in Figure 7. As including all AUC in this manuscript would make it extremely lengthy, we presented only one AUC for each classification. In Figure 6 and Figure 7, the AUC curves of qSOFA and SIRS were not in the group of extracted features as used in the original data. This is because they used the judging criteria set by the medical community to calculate the score. Thus, qSOFA and SIRS were the comparative procedures of our experiments. The accuracy rate and the Delong test for mortality prediction at 72 h and 28 d are listed in Table 1 and Table 2, respectively. The accuracy value is the average eight values from the four testing results.

### 3.4. Importance of Feature

During the process of accuracy testing, CNN + SoftMax produced the best results; however, it was unknown as to which baseline features were more important. To determine which features are most important, RF was used. Among those used in this study, RF is the only method capable of evaluating the importance of features, including RF itself. Table 3 and Table 4 list the feature importance for mortality prediction by RF at 72 h and 28 d, respectively. All four tests generate the results of K-fold cross validation.

## 4. Discussion

Since sepsis is diverse disease with high mortality rate, various ways of mortality prediction were developed. Biomarkers like procalcitonin, presepsin, CRP all played some role in sepsis mortality prediction, but the reported AUC of ROC curve varies from different settings or sepsis definition. In the same emergency department setting, Liu reported the AUC 0.658 for presepsin and AUC 0.679 for procalcitonin in predicting 28 d mortality [32]. Lee reported the AUC 0.76 for procalcitonin and 0.68 for CRP in predicting early mortality and the AUC 0.73 for procalcitonin and 0.64 for CRP in predicting late mortality [33]. These results were not promising compared to other ways in clinical use. Although many biomarkers display relevant correlation with the mortality of patients with sepsis, their time courses may be more reliable than absolute levels [34].

Compared to a single biomarker, the scoring system developed from the regression model could provide more feasible and cheap prediction methods. SIRS, SOFA score, qSOFA, MEDS (mortality in emergency department sepsis), NEWS (national early warning score) were used for this purpose in clinical practice and literature [35,36,37,38]. Lee reported an AUC 0.82, Zhao showed an AUC 0.776 and Hermans revealed an AUC 0.81 (95% CI = 0.73–0.88) for MEDS in predicting mortality [35,36]. Glouden reported an AUC 0.65 (95% CI = 0.61–0.68) for NEWS, 0.62 (95% CI = 0.59–0.66) for qSOFA in predicting in-hospital mortality [37]. Macdonald revealed an AUC 0.81 (95% CI = 0.74–0.88) for MEDS and an AUC 0.78 (95% CI = 0.71–0.87) for SOFA score in predicting sepsis related mortality [38]. The performance of MEDS was better than other scoring systems and biomarkers but it required both clinical presentation data and laboratory data to reach the accuracy [39]. Zhao combined MEDS and procalcitonin in predicting sepsis related mortality and increased the performance of AUC from 0.776 to 0.813.

In our study, we demonstrate that the accuracy rate of mortality prediction at both 72 h and 28 d of suspected infection in sepsis patients can be improved with machine learning. Among the different methods tested, we found that CNN + Softmax yielded the best predictions for sepsis-related mortality. Although machine learning is now used for various medical applications, very few studies used these algorithms in sepsis-related mortality prediction. In Taylor’s study, 500 clinical variables were used with the RF model to predict 28 days in-hospital sepsis-related mortality among 5278 ED visits [40]. With this, an area under the curve (AUC) of AUC 0.86 (95% CI = 0.82–0.90) was achieved, which is similar to our study that produced an AUC value of 0.89 (95% CI = 0.886–0.894). Taylor compared the traditional regression model and risk stratification score system and showed that they had a lower AUC than the RF model. In Rabias’s study, there were 34 clinical variables that were used with the RVM (relevance vector machine, a variant of SVM) model to predict severe sepsis-related mortality among 354 ICU patients [7]. He showed that RVM had AUC of 0.80, lower than our result (0.93 at 72 h and 0.90 at 28 days).

Through the feature visualization method, we ensured that the feature extraction abilities of CNN and AE were better than that of PCA. We determined whether features of the same group were concentrated together, and whether the features of different groups were separated.

The extracted feature visualizations show that CNN performed best because it segregated the surviving patients from the non-surviving patients. Features common to both surviving and non-surviving patients were centralized. The feature-extracted results of AE were better than those of PCA because the features of non-surviving patients were more centralized than for PCA, although the degrees of separation of surviving patient data were similar.

The feature extraction ability of the CNN was still the best in predicting mortality at 28 days. Although the features of surviving patients and non-surviving patients were closer than the case for mortality in 72 h, the features of AE and PCA were considerably worse. The CNN centralized the features of non-surviving patients. AE also centralized the non-surviving-patient features; however, the degree of centralization was less than CNN. The extracted results when predicting mortality within 28 days using PCA was worse than CNN. Therefore, in centralizing the same-labeled data or separating different labeled data, the ability of PCA was considerably inferior.

Certain AUC of the no feature extraction case with SoftMax were better than those using the CNN + SoftMax, yet the performance of the accuracy score demonstrated that results generated by CNN + SoftMax were the best. We believe that the accuracy score is more representative than AUC because AUC needs to set a prediction threshold for tuning. Setting the threshold immediately as 0.5 is a more accurate method for comparing the predictive ability of the algorithms.

AUCs of all methods were larger than 0.5 at 28 d. In most of the experiments, SoftMax demonstrated the best performance. The AUC at both 72 h and 28 d revealed that the best AUC score was obtained using CNN + SoftMax; however, the AUC curve is based on the calculation of each operation point on the regression. In terms of machine learning, it was more advantageous to have fewer handcrafted parameters. Although the AUC curve demonstrated the performance of various algorithms, its performance was based on multiple operating points.

Irrespective of the mortality prediction for 72 h or 28 d, predictions made with machine learning were more accurate than those made by either qSOFA or SIRS. Machine learning algorithms easily obtained an accurate result and improved both the treatment strategy and the mortality rate of suspected infected patients. Such methods ensured that the algorithm was considerably biased to the accurate rate of a single category. The most obvious type of partiality was the RF. The best accuracy rate was that of CNN + SoftMax. For more complex algorithms of feature extraction, the KNN, SVM, and RF trends were more accurate. Conversely, higher complexity of feature extractions resulted in higher accuracy rates for SoftMax. Irrespective of the feature extraction, SoftMax demonstrated the best performance. The SVM ranked second while recognizing the extracted features by the CNN.

Our results favored the use of a median value of 0.5 for the regression lines as the standard operating point for automatic classifiers. This comparison method can be considered the fairest and most reasonable. Regardless of which machine learning algorithm is used, it must have the ability to learn the operating points itself. For mortality prediction at 72 h, the accuracy rate of CNN + SoftMax was 89.02%; however, for mortality prediction at 28 d, the accuracy rate of this approach was reduced to 81.79%. This is acceptable because a mortality prediction at 28 d is inherently more difficult. The physiological state of a patient 28 d after diagnosis can be significantly different from the physiological state during the first examination. The longer the timeline, the more difficult it is to predict the mortality rate. Thus, the best classifier for mortality prediction at 28 d was SoftMax.

Irrespective of the type of feature extraction, or in the case where no feature extraction was used, the performance of SoftMax was the best and that of RF was the worst. While using SoftMax, the accuracy rate did not significantly differ. This demonstrated that the 28 d mortality prediction easily reaches its maximum limitation. Nevertheless, complex feature extraction is useful for other classifiers, particularly, the SVM and RF. The recognition abilities of SVM and RF were not equivalent to that of SoftMax. Therefore, increasing the strength of feature extraction optimizes the performance of a classifier. However, if the ability of the classifier is sufficient to appropriately classify the original features, then complex feature extraction does not provide an advantage. While using the SVM and RF as a classifier, we proposed using a CNN for feature extraction to increase the accuracy of the 28-d mortality prediction.

RF performance was inconsistent and was not improved by the use of complex feature extraction. The performance of the RF in mortality prediction at 28 d was similar to that at 72 h. The reason the experimental result for RF was so poor is that it could not effectively distinguish non-survivors from survivors. Thus, it can be inferred that the false positive rate was extremely high.

As the highest accuracy rate was obtained using CNN with a SoftMax layer, this algorithm was recommended for predicting the mortality of suspected infected patients in the ED. Such a design will make it easier for clinicians to immediately determine the condition of the patient.

CNN + SoftMax performed the best due to the shared weights and biases system. Through the shared weights and bias system, the calculation of the neurons was reduced. Regardless of the input feature dimensions, calculations of the neuron number times feature numbers were fixed by the shared weights and biases system. Unlike a traditional neural network, the calculation of neurons was not increased while the input dimension increased. Additionally, the pooling system further increased the performance of CNN + SoftMax, as it reduces the dimension of the extracted feature by the convolutional layer. Through this system, CNN can construct the importance information into a lower dimension feature. For a training model, the lower the dimension, the less gradient descent needs to be calculated. These systems further demonstrate that CNN + SoftMax has the best performance in our experiment. Other research experiments also show that CNN + SoftMax has a better performance than the other machine learning algorithms [41,42].

In our study, the most important feature extracted from RF was base excess (BE). Although it was not given much attention in our practice, one recent study showed that alactic base excess was associated with fluid balance, which is an important parameter in sepsis treatment [43]. Arayici showed that BE could be used to predict neonatal sepsis with promising sensitivity and specificity [44]. Another important feature is RDW, which has been well studied in various contexts; studies showed that it could be used to predict long-term outcomes in sepsis patients, irrespective of anemia [45]. Other important features, like solid tumors and shock episodes, were more familiar to clinical physicians.

The features that were extracted by CNN and AE could not be used to determine which baseline features were most important. Certain machine learning algorithms, such as RF, are capable of ranking different features according to their level of importance. Although their accuracy scores and AUC were lower than those of the CNN, observing these features may lead to other clinical applications.

Although our study demonstrated the good prediction ability of machine learning, it had several limitations. First, although the AUC of CNN + SoftMax is better than that of qSOFA and SIRS, and possibly other score systems as well, our model requires a computer for complex calculations, while the other methods involve calculations that can be easily performed by doctors. Second, CNN + SoftMax needs more clinical data to maintain its accuracy, while qSOFA and SIRS only require a few clinical data points. Thus, our method requires more time to produce accurate results, which in turn creates further delays to doctors and patients before they receive the information. As the use of computers in daily practice progresses, this problem may be resolved in the future. Third, in this study, we used mortality as the primary outcome. Although it is truly the final and worst outcome, and would not be influenced by the management decisions of patients, other bad outcomes like intensive care unit admission, or intubation, could provide more time for attending physicians to act on this disease.

## 5. Conclusions

In this study, three types of feature extraction processes and four types of classifications were implemented to predict the mortality of suspected infected patients in a hospital emergency department. The accuracy rates of machine learning methods were higher than those for existing medical methods, i.e., SIRS and qSOFA. Among them, the performance of CNN with SoftMax exhibited the highest accuracy with a rate of 89.02% for mortality within 72 h and 81.79% for mortality within 28 d.

## Figures and Tables

**Table 1:** AUC with 95% confidence interval and the accuracy rate of various methods in predicting 72-h mortality and compared with CNN plus SoftMax by Delong test.

Algorithms	AUC	SE	95%CI	Compared with CNN + SoftMax	Acc (%)
SIRS	0.67	0.0101	0.67–0.68	*p* < 0.0001	59.43
qSOFA	0.74	0.0101	0.73–0.74	*p* < 0.0001	67.27
RF	0.89	0.0067	0.88–0.89	*p* < 0.0001	62.56
KNN	0.83	0.0087	0.83–0.84	*p* < 0.0001	77.31
SVM	0.93	0.0044	0.92–0.93	*p* < 0.0001	74.33
SoftMax	0.91	0.0052	0.91–0.92	*p* < 0.0001	82.73
PCA + RF	0.90	0.0059	0.90–0.91	*p* < 0.0001	62.62
PCA + KNN	0.88	0.0071	0.88–0.89	*p* < 0.0001	81.67
PCA + SVM	0.91	0.0055	0.90–0.91	*p* < 0.0001	78.91
PCA + SoftMax	0.92	0.0050	0.92–0.93	*p* < 0.0001	83.48
AE + RF	0.77	0.0064	0.76–0.77	*p* < 0.0001	63.52
AE + KNN	0.92	0.0053	0.91–0.92	*p* < 0.0001	80.64
AE + SVM	0.85	0.0086	0.85–0.85	*p* < 0.0001	78.76
AE + SoftMax	0.93	0.0042	0.92–0.93	*p* < 0.0001	84.17
CNN + RF	0.87	0.0069	0.87–0.88	*p* < 0.0001	61.03
CNN + KNN	0.86	0.0069	0.85–0.86	*p* < 0.0001	81.73
CNN + SVM	0.92	0.0047	0.92–0.92	*p* < 0.0001	84.96
**CNN + SoftMax**	**0.94**	**0.0043**	**0.94–0.94**	**None**	**87.01**

Abbreviation: SIRS, systemic inflammatory response syndrome; qSOFA, quick sepsis-related organ failure assessment; RF, random forest; KNN, K nearest neighbor; SVM, support vector machine; PCA, principal component analysis; AE, autoencoder; CNN, convolutional neural network; AUC, area under the curve; SE standard error; CI, confidence interval; Acc, accuracy.

**Table 2:** AUC with 95% confidence interval and the accuracy rate of various methods in predicting 28-days mortality and compared with CNN plus SoftMax by Delong test.

Algorithms	AUC	SE	95%CI	Compared with CNN + SoftMax	Acc (%)
SIRS	0.59	0.0063	0.59–0.60	*p* < 0.0001	59.43
qSOFA	0.68	0.0061	0.67–0.69	*p* < 0.0001	67.27
RF	0.89	0.0032	0.89–0.89	*p* < 0.0001	62.56
KNN	0.84	0.0047	0.83–0.84	*p* < 0.0001	77.31
SVM	0.90	0.0031	0.89–0.90	*p* < 0.0001	74.33
SoftMax	0.88	0.0034	0.90–0.89	*p* < 0.0001	82.73
PCA + RF	0.89	0.0034	0.89–0.89	*p* < 0.0001	62.62
PCA + KNN	0.84	0.0050	0.84–0.85	*p* < 0.0001	81.67
PCA + SVM	0.89	0.0033	0.88–0.89	*p* < 0.0001	78.91
PCA + SoftMax	0.91	0.0031	0.90–0.91	*p* < 0.0001	83.48
AE + RF	0.84	0.0037	0.83–0.84	*p* < 0.0001	63.52
AE + KNN	0.81	0.0042	0.81–0.82	*p* < 0.0001	80.64
AE + SVM	0.89	0.0033	0.89–0.90	*p* < 0.0001	78.76
AE + SoftMax	0.90	0.0032	0.89–0.90	*p* < 0.0001	84.17
CNN + RF	0.90	0.0032	0.90–0.91	*p* < 0.0001	61.03
CNN + KNN	0.86	0.0040	0.85–0.86	*p* < 0.0001	81.73
CNN + SVM	0.92	0.0027	0.91–0.92	*p* < 0.0001	84.96
**CNN + SoftMax**	**0.92**	**0.0027**	**0.92–0.92**	**None**	**87.01**

Abbreviation: SIRS, systemic inflammatory response syndrome; qSOFA, quick sepsis-related organ failure assessment; RF, random forest; KNN, K nearest neighbor; SVM, support vector machine; PCA, principal component analysis; AE, autoencoder; CNN, convolutional neural network; AUC, area under the curve; SE standard error; CI, confidence interval; Acc, accuracy.

**Table 3:** Feature importance of 72 h mortality prediction by Random Forest (RF) (%).

Test 1		Test 2		Test 3		Test 4	
Feature	Importance	Feature	Importance	Feature	Importance	Feature	Importance
BE	35.60	BE	39.50	BE	33.59	BE	36.50
Shock episode	12.89	Shock episode	11.86	Shock episode	13.89	Shock episode	13.00
GCS (V)	7.62						
~ Lower than 5% ignored ~							

Abbreviation: RF, random forest; BE, base excess; GCS (V), Glasgow Coma Scale- Verbal response.

**Table 4:** Feature importance of 28 d mortality prediction by RF (%).

Test 1		Test 2		Test 3		Test 4	
Feature	Importance	Feature	Importance	Feature	Importance	Feature	Importance
BE	20.39	BE	23.38	BE	19.88	BE	20.29
RDW-SD	9.07	Solid tumor	6.00	RDW-SD	10.11	RDW-CV	8.55
RDW-CV	5.53	RDW-CV	5.80			Solid tumor	5.55
Solid tumor	5.35	RDW-SD	5.43			RDW-SD	5.54
~ Lower than 5% ignored ~							

Abbreviation: RF, random forest; BE, base excess; RDW-SD, Red cell distribution width standard deviation; RDW-CV, Red cell distribution width coefficient of variation.

## Appendix A

**Table A1:** Baseline features for machine learning (first reading on admission).

No.	Clinical Variables	Survived	Non-Surviving	*p*	Miss (%)
1	Blood Pressure	139.76 ± 32.48	121.17 ± 43.80	<0.001	0
2	Triage			<0.001	0
	1	6.3%	27.3%		
	2	33.4%	40.7%		
	3	57.0%	29.9%		
	4	3.1%	2.0%		
	5	0.2%	0.1%		
3	GCS (E)			<0.001	0
	1	2.1%	14.2%		
	2	2.5%	8.1%		
	3	3.4%	7.9%		
	4	92.0%	69.8%		
4	GCS (V)			<0.001	0
	1	8.4%	27.5%		
	2	3.5%	9.3%		
	3	1.4%	3.0%		
	4	3.0%	5.7%		
	5	83.7%	54.5%		
5	GCS (M)			<0.001	0
	1	0.9%	10.2%		
	2	1.1%	4.4%		
	3	2.8%	6.8%		
	4	4.8%	10.9%		
	5	5.5%	11.6%		
	6	84.9%	56.2%		
6	WBC	11.32 ± 8.36	14.29 ± 16.95	<0.001	0.017
7	Hb	12.01 ± 2.39	10.74 ± 2.61	<0.001	0.008
	Seg	76.98 ± 13.37	77.96 ± 16.84	<0.001	0.064
9	Lymph	14.81 ± 10.67	12.02 ± 12.57	<0.001	0.067
10	PT-INR	1.21 ± 0.55	1.58 ± 0.93	<0.001	21.94
11	BUN	24.76 ± 24.33	45.21 ± 37.56	<0.001	0
12	Cr	1.47 ± 1.79	2.29 ± 2.30	<0.001	0
13	Bil	2.42 ± 3.74	6.13 ± 8.51	<0.001	20.16
14	AST	73.29 ± 258.18	258.10 ± 1099.87	<0.001	16.46
15	ALT	47.24 ± 129.98	105.73 ± 364.09	<0.001	0
16	Troponin I	0.33 ± 3.20	1.43 ± 8.52	<0.001	24.21
17	pH	7.40 ± 0.11	7.33 ± 0.18	<0.001	22.79
18	HCO3	23.41 ± 6.49	20.49 ± 8.01	<0.001	22.79
19	Atypical lymphocyte	0.078 ± 0.51	0.18 ± 0.66	<0.001	0.067
20	Promyelocyte	0.0071 ± 0.45	0.044 ± 1.23	<0.001	0.067
21	Metamyelocyte	0.11 ± 0.51	0.55 ± 1.60	<0.001	0.067
22	Myelocyte	0.15 ± 0.71	0.61 ± 1.53	<0.001	0.001
23	Sodium ion	135.48 ± 5.60	134.51 ± 8.67	<0.001	0.067
24	Potassium ion	3.91 ± 0.71	4.30 ± 1.14	<0.001	0
25	Albumin	2.99 ± 0.72	2.55 ± 0.63	<0.001	25.79
26	Sugar	163.16 ± 106.24	192.04 ± 167.08	<0.001	15.10
27	RDW-SD	46.46 ± 7.54	53.69 ± 11.54	<0.001	0.012
28	MCV	88.44 ± 8.14	90.35 ± 9.26	<0.001	0.011
29	RDW-CV	14.49 ± 22.24	16.50 ± 3.27	<0.001	0.012
30	Base excess	−1.12 ± 6.44	−5.08 ± 9.15	<0.001	22.79
31	MCH	29.53 ± 3.16	29.91 ± 3.35	<0.001	0.010
32	MCHC	33.36 ± 1.39	33.11 ± 1.71	<0.001	0.011
33	MAP	101.14 ± 26.83	88.25 ± 32.54	<0.001	0.011
34	RR	19.58 ± 2.77	20.25 ± 6.04	<0.001	0
35	Temperature	37.37 ± 1.26	36.46 ± 4.21	<0.001	0.001
36	Heart rate	99.90 ± 23.00	102.66 ± 33.95	<0.001	0
37	Age	61.05 ± 18.11	68.52 ± 15.02	<0.001	0
38	Sex (male%)	52.6%	61.3%	<0.001	0
39	qSOFA Score			<0.001	0
	0	69.7%	33.1%		0
	1	23.7%	38.1%		0
	2	6.0%	23.9%		0
	3	0.6%	4.8%		0
40	Shock episode	2.5%	26.8%	<0.001	0
41	Liver cirrhosis	6.9%	17.6%	<0.001	0
42	DM	25.2%	26.4%	0.029	0
43	CRF	10.3%	28.1%	<0.001	0
44	CHF	4.5%	9.1%	<0.001	0
45	CVA	8.6%	12.5%	<0.001	0
46	Solid tumor	18.0%	43.6%	<0.001	0
47	RI	66.0%	48.9%	<0.001	0
48	UTI	21.1%	15.7%	<0.001	0
49	Soft tissue infection	13.7%	4.7%	<0.001	0
50	Intra-abdominal infection	11.2%	10.6%	0.141	0
51	Other infection	35.7%	33.4%	<0.001	0
52	Bacteremia	8.1%	16.5%	<0.001	0
53	Antibiotic used within 24 h	77.9%	85.5%	<0.001	0

Abbreviation: GCS (E), Glasgow Coma Scale eye opening; GCS (V), Glasgow Coma Scale verbal response; GCS (M), Glasgow Coma Scale motor response; WBC, white blood cell count; Hb, hemoglobin; Seg, segment; Lymph, lymphocyte; PT-INR, prothrombin time international normalized ratio; BUN, Blood urea nitrogen; Cr, creatinine; Bil, bilirubin; AST, glutamic-pyruvic transaminase; ALT, alanine aminotransferase; pH, pondus hydrogenii; HCO3, hydrogen carbonate Ion; RDW-SD, red cell distribution width standard deviation; MCV, mean corpuscular volume; RDW-CV, red cell distribution width coefficient of variation; MCH, mean corpuscular hemoglobin; MCHC, mean corpuscular hemoglobin concentration; MAP, mean arterial pressure; RR, respiratory rate; DM, diabetes mellitus; CRF, chronic renal failure; CVA, congestive heart failure; RI, Respiratory infection; UTI, urinary tract infection.
